# Two new aromatic polyketides from a sponge-derived *Fusarium*

**DOI:** 10.3762/bjoc.15.289

**Published:** 2019-12-09

**Authors:** Mada Triandala Sibero, Tao Zhou, Keisuke Fukaya, Daisuke Urabe, Ocky K Karna Radjasa, Agus Sabdono, Agus Trianto, Yasuhiro Igarashi

**Affiliations:** 1 Department of Marine Science, Faculty of Fisheries and Marine Science, Diponegoro University, Tembalang Campus, St. Prof. Soedarto SH, Semarang, 50275 Central Java, Indonesia; 2 Marine Science Techno Park, Diponegoro University, Teluk Awur Campus, St. Undip, Jepara District, Central Java, Indonesia; 3 Biotechnology Research Center, Toyama Prefectural University, 5180 Kurokawa, Imizu, Toyama 939-0398, Japan

**Keywords:** aromatic polyketide, *Fusarium*, marine fungus, secondary metabolite, sponge

## Abstract

In our natural product screening program from marine fungi, two new aromatic polyketides karimunones A (**1**) and B (**2**) and five known compounds (**3**–**7**) were isolated from sponge-associated *Fusarium* sp. KJMT.FP.4.3 which was collected from an Indonesian sponge *Xestospongia* sp. The structures of these compounds were determined by the analysis of NMR and MS spectroscopic data. The NMR assignment of **1** was assisted by DFT-based theoretical chemical shift calculation. Compound **2** showed antibacterial activity against multidrug resistant *Salmonella enterica* ser. Typhi with a MIC of 125 µg/mL while **1** was not active.

## Introduction

Marine organisms have been known as a potential source of prospective bioactive compounds, and sponges are particularly emphasized as the most promising source among all marine invertebrates [1–2]. However, the collection of sponges in massive amounts leads to environmental disturbance since marine sponges play a key role in building coral reefs [3–4]. As a filter feeder, sponges host an enormous amount of microorganisms including algae, bacteria, actinomycetes, and fungi [5–7]. Many of these microorganisms produce structurally unique secondary metabolites with various biological activities [8–9]. Specifically, sponge-associated fungi are attracting substantial attention because of their high capability of producing a wide range of bioactive compounds [5,10–11]. As a tropical country, Indonesia is known as the second most prospective country for new natural products from marine resources owing to its high biodiversity [12]. Thus far, sponges have also been utilized as the most productive source of novel compounds in Indonesia. According to the latest review by Hanif et al. [13], ca. 500 marine natural products were obtained from Indonesian sponges from January 1970 to December 2017, while less than 50 compounds were reported from marine fungi during the same period. Furthermore, only a few compounds were isolated from fungal symbionts in Indonesian sponges to date [14–15]. This is strongly indicating that sponge-associated fungi from Indonesia are still underevaluated. Therefore, exploration of novel compounds from fungi associating with Indonesian sponges is currently a major subject in our research group [16–18].

**Figure 1:**
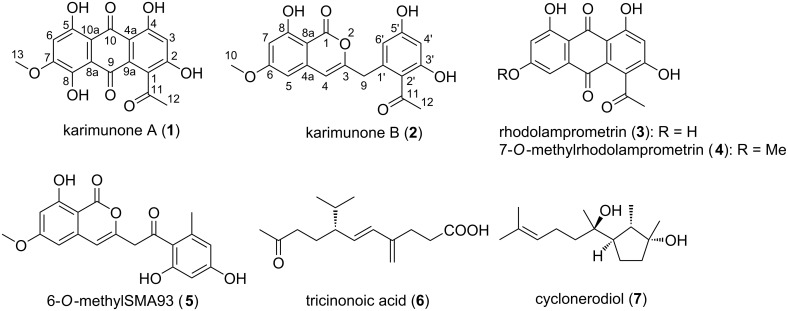
Structures of compounds **1**–**7**.

Along this line of study, *Fusarium* sp. KJMT.FP.4.3 was isolated from a sponge *Xestospongia* sp. collected in Karimunjawa National Park, Indonesia. This fungus produces a violet pigment in the mycelium and secretes pink to red pigments into the broth medium. Comprehensive chemospectroscopic analysis of the culture extract using HPLC/UV led to the isolation of two new aromatic polyketides, karimunones A (**1**) and B (**2**) along with five known compounds, rhodolamprometrin (**3**) [19], 7-*O*-methylrhodolamprometrin (**4**) [20], 6-*O*-methylSMA93 (**5**) [21], tricinonoic acid (**6**) [22], and cyclonerodiol (**7**) [23] (Figure 1). We herein describe the isolation, structure determination, and biological activity of **1** and **2**.

## Results and Discussion

A fungal strain was isolated from a sponge *Xestospongia* sp. collected in Karimunjawa National Park, Central Java, and identified as a member of *Fusarium* on the basis of sequence similarity of the internal transcribed spacer (ITS) domain to *Fusarium oxysporum* strains in the DNA database. HPLC/UV-guided purification of the secondary metabolites from this strain led to the isolation of two new polyketides, karimunones A (**1**) and B (**2**), together with five known compounds (**3**–**7**, Figure 1).

Compound **1** was obtained as a red powder. TOF-HRESIMS analysis gave a deprotonated molecule [M − H]^−^ at *m*/*z* 343.0452 corresponding to a molecular formula of C_17_H_12_O_8_ (calcd for C_17_H_11_O_8_, 343.0459). Combination of ^13^C NMR and HSQC analytical data revealed the presence of 17 carbons assignable to three carbonyl carbons (δ_C_ 179.8, 186.6, and 201.3), twelve aromatic sp^2^ carbons (five are oxygenated and two are proton-bearing), and two methyl groups (Table 1). In the ^1^H NMR spectrum, all resonances were observed as a singlet peak and were assigned to two methyls (δ_H_ 2.42 and 3.95), two aromatic methines (δ_H_ 6.67 and 6.95), and three exchangeable protons (δ_H_ 12.57, 12.65, 12.70). The UV spectrum of **1** with the absorption bands at 234, 266, 313, 499, and 533 nm was closely similar to that for 1-acetyl-2,4,5,7,8-pentahydroxyanthraquinone from a fungus *Geosmithia* [24], suggesting the presence of a common chromophore in **1**.

**Table 1:** NMR spectroscopic data for karimunone A (**1**) in DMSO-*d*_6_.

position	δ_C_, type^a^	δ_H_, mult^b^	HMBC^c^

1	126.3, C		
2	161.5, C		
3	108.5, CH	6.67, s	1, 2, 4, 4a
4	163.8, C		
4a	108.5, C		
5	159.1, C		
6	107.9, CH	6.95, s	5, 7, 8, 10a
7	157.1, C		
8	149.7, C		
8a	111.9, C		
9	179.8, C		
9a	130.6, C		
10	186.6, C		
10a	104.5, C		
11	201.3, C		
12	30.9, CH_3_	2.42, s	1, 11
13	56.9, CH_3_	3.95, s	7
4-OH		12.57, s	3, 4, 4a
OH		12.65, br.s	
OH		12.70, br.s	

^a^Recorded at 125 MHz (reference δ_C_ 39.5). ^b^Recorded at 500 MHz (reference δ_H_ 2.50). ^c^HMBC correlations are from proton(s) stated to the indicated carbon.

**Figure 2:**
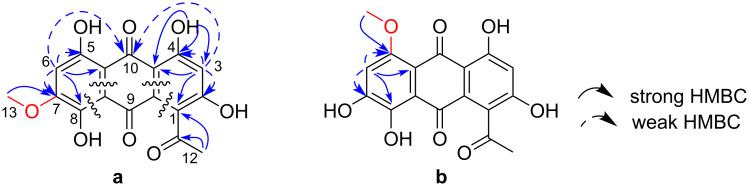
Key HMBC correlations and two possible structures (**a** and **b**) for karimunone A (**1**).

Two highly substituted benzene rings were assembled by analysing the HMBC correlation data (Table 1, Figure 2). Strong correlations from H3 to C1 and C4a indicated *meta*-relationships for C1, C3, and C4a, while weak correlations from H3 to C2 and C4 suggested *ortho*-positioning of C2 and C4 to C3. An acetyl group at C1 was confirmed by the HMBC correlations from H12 to C11 and C1. HMBC correlations from an exchangeable proton at δ_H_ 12.57 (4-OH) to C3, C4, and C4a were also supportive of the substitution pattern in this benzene ring. Strong correlations from H6 to C8 and C10a implied the *meta*-relationship of C8 and C10a to C6, and similarly the *ortho*-relationship of C5 and C7 to C6 were deduced from the weak correlations from H6 to C5 and C7. In addition, deshielded resonances for C5, C7, and C8 indicated that these carbons were oxygenated. The methyl protons of the methoxy group (H13) had an HMBC correlation to a carbon at δ_C_ 157.1 but it was not possible to determine the site of methoxy substitution only from the available HMBC data. Four-bond correlations detected from H3 and H6 to C10 indicated a carbonyl-bridge between C4a and C10a. Although no further long-range correlations were available, two aromatic carbons (C8a, C9a) and one carbonyl carbon (C9) were placed between the two rings to complete the anthraquinone skeleton in consideration of the UV spectrum and the molecular formula, providing two possible structures **a** and **b** for **1** (Figure 2): the methoxy group is positioned at C7 in structure **a** and at C5 in structure **b**.

In order to eliminate one of the two possible structures for **1**, the experimental chemical shifts of **1** were compared with the NMR chemical shifts calculated for structures **a** and **b** at mPW1PW91/6-31G+(d,p)-PCM(DMSO) level of theory. Since only five resonances including one exchangeable phenolic proton were available for ^1^H chemical shifts, ^13^C chemical shifts were used for comparison. The theoretical ^13^C NMR data of structure **a** showed better agreement with the experimental ^13^C NMR data of **1**. Specifically, the chemical shift difference between the experimental and theoretical values for the carbons spatially close to the methoxy group (C5, C6, C7, C8, C8a, C9, C10, and C10a) were significantly smaller for structure **a** than structure **b** (Table 2). Then, DP4+ was used to quantify this calculated result [25]. DP4+ probability analysis provides reliable guidance on the correct structure among several possible isomers. The developers recommend to use the combination of ^1^H and ^13^C data for enhancement of DP4+ performance. In this study, the DP4+ probability using only ^1^H data or only ^13^C data gave an inconsistent result, but the DP4+ probability using both ^1^H and ^13^C data supported the structure **a**, in which the methoxy group was present at C7, as a correct structure for **1** (100% for structure **a** and 0.0% for structure **b**, Table 2).

**Table 2:** DFT-calculated NMR chemical shifts of two possible structures **a** and **b** for karimunone A (**1**) at the mPW1PW91/6-31G+(d,p)-PCM(DMSO) level.

	karimunone A (**1**)		structure **a**		structure **b**		structure **a**	structure **b**

position	δ_C(exp)_	δ_H(exp)_	δ_C(calc)_	δ_H(calc)_	δ_C(calc)_	δ_H(calc)_	|δ_C(exp)_ – δ_c(calc)_|	|δ_C(exp)_ – δ_c(calc)_|

1	126.3		122.8		121.7		3.5	4.6
2	161.5		156.7		155.6		4.8	5.9
3	108.5	6.67	107.5	6.89	108.1	6.90	1.0	0.4
4	163.8		162.3		162.4		1.5	1.4
4a	108.5		110.2		110.9		1.7	2.4
5	159.1		157.1		155.2		2.0	3.9
6	107.9	6.95	106.6	6.93	104.1	7.27	1.3	3.8
7	157.1		155.4		151.3		1.7	5.8
8	149.7		148.3		142.0		1.4	7.7
8a	111.9		111.9		114.7		0.0	2.8
9	179.8		183.4		185.7		3.6	5.9
9a	130.6		133.8		132.1		3.2	1.5
10	186.6		182.8		181.1		3.8	5.5
10a	104.5		104.3		109.2		0.2	4.7
11	201.3		204.8		204.7		3.5	3.4
12	30.9	2.42	33.7	2.58	33.8	2.58	2.8	2.9
13	56.9	3.95	56.6	4.08	55.9	4.05	0.3	1.0
4-OH		12.57		12.53^*^		13.51^*^		

			structure **a**		structure **b**			

DP4+ (^1^H data)			1.5%		98.5%			
DP4+ (^13^C data)			100.0%		0.0%			
DP4+ (^1^H and ^13^C data)			100.0%		0.0%			

^*^Exchangeable signals are not included for evaluation.

**Figure 3:**
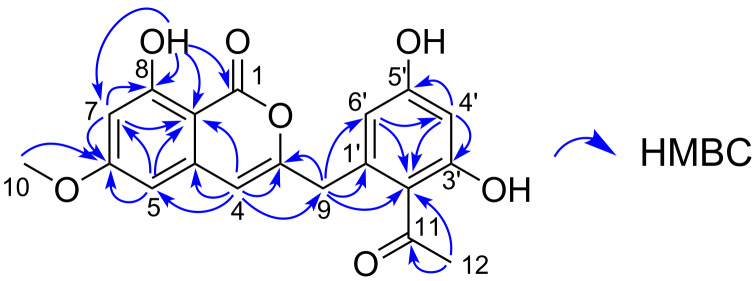
Key HMBC correlations for karimunone B (**2**).

Compound **2** was also isolated as a reddish powder. TOF-HRESIMS analysis gave an [M − H]^−^ ion peak at *m*/*z* 355.0823 corresponding to a molecular formula of C_19_H_16_O_7_ (calcd for C_19_H_15_O_7_, 355.0818). The IR spectrum showed a strong absorption band at 1683 cm^−1^, indicating the presence of carbonyl functionality. The ^13^C NMR exhibited 19 carbon signals that could be assigned to eleven nonprotonated sp^2^ carbons (seven are oxygenated), five sp^2^ methine carbons, one methylene carbon, and two methyl carbons from HSQC spectral data (Table 3). Mutual HMBC correlations between two aromatic methines H5 and H7 and their correlations to C8a established their *meta*-relationship in a benzene ring system. A methoxy group was placed at C6 on the basis of HMBC correlations from H5, H7, and H10 to C6. A sharp singlet resonance at δ_H_ 10.94 was deduced to be a hydrogen-bonded phenolic proton that showed HMBC correlations to C7, C8, and C8a, completing the tetrasubstituted benzene ring with a hydroxy and a methoxy substituent. To this ring were connected a carbonyl carbon C1 at C8a and a three-carbon fragment C4–C3–C9 at C4a by the HMBC correlations from 8-OH to C1 and H4 to C3, C4a, C5, C8a, and C9, respectively. Another tetrasubstituted benzene ring was deduced also from the HMBC analysis. Mutual HMBC correlations of two aromatic protons H4’ and H6’ and their correlations to C2’ revealed the *meta*-relationship among C2’, C4’ and C6’. Shielded chemical shifts of C2’, C4’, and C6’ and deshielded chemical shifts of C3’ and C5’ suggested the presence of hydroxy groups at C3’ and C5’. HMBC correlations from H12 to C2’ and C11 as well as a highly deshielded carbon chemical shift for C11 (δ_C_ 203.5) indicated the acetyl substituent at C2’. This benzene ring was then connected to the methylene carbon C9 by the correlations from H9 to C1’, C2’, and C6’. Finally, consideration of the deshielded chemical shift of C3 and the molecular formula allowed the linkage between C1 and C3 via an oxygen atom to complete the structure of **2** (Figure 3).

**Table 3:** NMR spectroscopic data for karimunone B (**2**) in CDCl_3_.

position	δ_C_, type^a^	δ_H_, mult.^b^	HMBC^c^

1	166.0, C		
3	155.9, C		
4	105.9, CH	5.98, s	3, 4a, 5, 8a, 9
4a	138.8, C		
5	102.0, CH	6.27, s	4, 6, 7, 8a
6	167.3, C		
7	101.2, CH	6.48, s	5, 6, 8, 8a
8	163.9, C		
8a	99.9, C		
9	39.8, CH_2_	4.07, s	3, 4, 1', 2', 6'
10	56.0, CH_3_	3.85, s	6
11	203.5, C		
12	32.3, CH_3_	2.61, s	11, 2'
1'	138.6, C		
2'	115.7, C		
3'	166.8, C		
4'	103.7, CH	6.41, s	2', 3', 5', 6'
5'	161.5, C		
6'	112.9, CH	6.34, s	2', 4', 5', 9
8-OH		10.94, s	1, 6, 7, 8, 8a

^a^Recorded at 125 MHz (reference δ_C_ 77.0). ^b^Recorded at 500 MHz (reference δ_H_ 7.27). ^c^HMBC correlations are from proton(s) stated to the indicated carbon.

Antibacterial activities of compounds **1–7** were tested against clinical pathogen *Salmonella enterica* ser. Typhi strain MDR from General Hospital Dr. Kariadi, Semarang, Indonesia. Compounds **2–7** were weakly active with MIC values of 125 µg/mL, while **1** was not active in the same concentration range.

## Conclusion

In summary, our chemical analysis of a sponge-derived fungus of the genus *Fusarium* led to the identification of two new and three known aromatic polyketides (**1** and **2**, and **3**–**5**, respectively) and two sesquiterpenes (**6** and **7**). Although secondary metabolites of *Fusarium* were extensively studied in the past [26–28], it is still possible to obtain additional new compounds, implying the existence of unstudied *Fusarium* species capable of producing unknown metabolites in marine symbiotic environments. Interestingly, **4** and its 5-*O*-methylated congener were previously reported from the Australian marine crinoid *Comatula rotalaria* [20]. Compound **4**, an 8-deoxy congener of **1**, is presumably a biosynthetic precursor for **1**. Our finding of **1**, **3**, and **4** from a sponge-associated fungus is suggestive of the production of these anthraquinones by symbiotic fungi in crinoids. The 7-hydroxy congener of **1** is known as a metabolite of the fungus *Geosmithia* [24]. Plant metabolites, feralolide and its glycoside, possessing the same skeleton as **2** were reported from the medicinal herb *Aloe vera* [29], but this carbon skeleton is novel as a fungal secondary metabolite.

## Experimental

### General experimental procedures

NMR spectra were recorded on a Bruker AVANCE II 500 spectrometer (Bruker Biospin K. K., Yokohama, Japan) and mass spectra were measured on a Bruker micrOTOF (Bruker Daltonics K. K., Yokohama, Japan). IR spectra were recorded on a Shimadzu FT-IR-300 spectrophotometer (Shimadzu Corp., Kyoto, Japan) and UV spectra on a Shimadzu UV-1800 (Shimadzu Corp., Kyoto, Japan).

### Microorganism

*Fusarium* sp. KJMT.FP.4.3 was isolated from a sponge *Xestospongia* sp. collected from Karimunjawa National Park, Indonesia with the permission number 1096/T.34/TU/SIMAKSI/7/2017. This fungus was isolated using a surface sterilization method [14,30]. Strain KJMT.FP.4.3 was identified as *Fusarium* on the basis of the gene sequence analytical data of the ITS domain of partial 18S rRNA gene and 28S rRNA gene. Strain KJMT.FP.4.3 showed 99.7% similarity of ITS domain of partial 18S rRNA gene and 28S rRNA gene (555 nucleotides, GeneBank accession number MK393925.1) to *Fusarium oxysporum* strain NZZCDHL (549 nucleotides, GeneBank accession number KU939031.1) and 99.5% similarity of ITS domain of partial 18S rRNA gene and 28S rRNA gene to *Fusarium oxysporum* isolate DG-2 (548 nucleotides, GeneBank accession number MK429839.1).

### Fermentation

Strain KJMT.FP.4.3 cultured on PDA agar was inoculated into 500 mL K-1 flasks each containing V22 seed medium consisting of soluble starch 1%, glucose 0.5%, NZ-case (Sigma-Aldrich, Co., LLC.) 0.3%, yeast extract (Kyokuto Pharmaceutical Industrial, Co., Ltd.) 0.2%, Tryptone (Difco Laboratories) 0.5%, K_2_HPO_4_ 0.1%, MgSO_4_·7H_2_O 0.05%, and CaCO_3_ 0.3% (pH 7.0). The flasks were shaken at 30 °C for 4 days on a rotary shaker (200 rpm). The seed culture (3 mL) was transferred into 500 mL K-1 flasks each containing 100 mL of A3M production medium (pH 7.0) consisting of 2.0% soluble starch, 0.5% glucose, 2.0% glycerol, 0.3% yeast extract, 1.5% Pharmamedia (Traders Protein), and 1% Diaion HP-20 (Mitsubishi Chemical Co.). The inoculated flasks were placed on a rotary shaker (200 rpm) at 30 °C for 7 days.

### Extraction and isolation

Extraction and isolation of secondary metabolites from strain KJMT.FP.4.3 were carried out in a similar manner as described previously [31]. At the end of the fermentation period, 50 mL of 1-butanol was added to each flask, and the flasks were shaken for 1 h. The mixture was centrifuged at 6000 rpm for 10 min and the organic layer was separated from the aqueous layer containing the mycelium. Evaporation of the solvent gave 3.37 g of crude extract from 1.5 L of culture. The crude extract (3.37 g) was subjected to silica gel column chromatography with a step gradient of CHCl_3_/MeOH 1:0, 20:1, 10:1, 4:1, 2:1, 1:1, and 0:1 (v/v). Fraction 3 (10:1) was concentrated to provide 0.97 g of brown oil, which was further purified by reversed-phase ODS column chromatography with a gradient of MeCN–H_2_O 2:8, 3:7, 4:6, 5:5, 6:4, 7:3, and 8:2 (v/v). Fraction 5 (6:4) was evaporated and the remaining aqueous solution was extracted with EtOAc. After drying with anhydrous Na_2_SO_4_, the organic layer was concentrated to dryness. The residual solid (53 mg) was applied to the preparative HPLC (Cosmosil Cholester Packed Column, 10 × 250 mm, Nacalai Tesque) using a linear gradient of 40 to 70% MeCN in 0.1% HCO_2_H over 25 min at a flow rate of 4 mL/min, yielding karimunone A (**1**, 7.3 mg, *t*_R_ 15.6 min) and B (2, 4.2 mg, *t*_R_ 12.0 min), together with 7-*O*-methylrhodolamprometrin (**4**, 7.2 mg, *t*_R_ 17.4 min), *O*-methylSMA93 (**5**, 7.4 mg, *t*_R_ 13.2 min), tricinonoic acid (**6**, 3.7 mg, *t*_R_ 13.1 min), and cyclonerodiol (**7**, 5.4 mg, *t*_R_ 11.3 min) after evaporation and extraction with EtOAc. Rhodolamprometrin (**3**, 4.0 mg, *t*_R_ 15.2 min) was isolated from fraction 4 of ODS chromatography (5:5), which was derived from fraction 3 of silica gel fractionation (10:1), using a linear gradient of 40 to 70% MeCN in 0.1% HCO_2_H over 25 min at a flow rate of 4 mL/min. Physicochemical data including ^1^H and ^13^C NMR spectroscopic data of compounds **3**–**7** were in good accordance with those described in the literatures [19–23].

Karimunone A (**1**): red powder; UV (MeOH) λ_max_ (log ε) 234 (3.44), 266 (3.28), 313 (3.04), 499 (2.81), 533 (2.68) nm; IR ν_max_ 3111, 2926, 1712, 1679, 1601 cm^−1^; see Table 1 for ^1^H NMR and ^13^C NMR data; TOF-HRESIMS [M − H]^−^ *m*/*z* 343.0452 (calcd for C_17_H_11_O_8_, 343.0459).

Karimunone B (**2**): red powder; UV (MeOH) λ_max_ (log ε) 244 (3.49), 277 (2.97), 324 (2.85) nm; IR ν_max_ 3100, 1683, 1615 cm^−1^; see Table 3 for ^1^H NMR and ^13^C NMR data; TOF-HRESIMS [M − H]^−^ *m*/*z* 355.0823 (calcd for C_19_H_15_O_7_, 355.0818).

### Computational procedure

#### General information

Conformational search was performed with MacroModel version 12.1 in the Maestro 11.7 software package [32–33]. All DFT-based calculations were performed with the Gaussian 16 Rev B.01 program [34]. A part of these computations were conducted using a Fujitsu PRIMERGY CX400 multi-node server (Information Technology Center of Nagoya University). Molecular structures were visualized using Maestro 11.7 software package. DP4+ analysis was performed with the Excel spreadsheet [25] made by Sarotti et al. Cartesian coordinates of the structures described in this paper are included in Supporting Information File 1.

#### Computational search and NMR calculations of structures **a** and **b**

The conformational search on structure **a** began by applying 100,000 steps of the Monte-Carlo Multiple Minimum (MCMM) method with PRCG energy minimization using the OPLS3e force field (gas phase) to obtain 42 conformational isomers within 35 kcal/mol from the minimum energy conformer. The next optimizations were performed at the M06-2X/6-31G(d) level of theory and solvation effects were included using the PCM solvation model (DMSO). Frequency calculations were carried out at the same level of theory to confirm the absence of imaginary frequencies and to obtain thermal corrections to the Gibbs free energies at 1 atm, 298.15 K. The duplicate structures with RMSDs of 0.01 Å were removed. Single-point energies were calculated at the M06-2X/6-311+G(d,p) level of theory and solvation effects were included using the PCM solvation model (DMSO). The NMR chemical shifts were simulated by GIAO method at the mPW1PW91/6-31G+(d,p)-PCM(DMSO) level of theory. The chemical shifts (δ_calc_) were calculated using tetramethylsilane (TMS) as a reference standard according to δ_calc_ = σ_0_ − σ_x_, where σ_x_ is the Boltzmann-averaged shielding tensor of the most stable 4 conformers within 3.5 kcal/mol and σ_0_ is the shielding tensor of TMS calculated at the same level of theory with σ_x_. The NMR shifts of structure **b** was similarly calculated using 50 structures as the OPLS3e-minimized structures and 4 structures as the DFT-optimized structures for the NMR calculation.

## Supporting Information

File 1 Copies of UV, IR, and NMR spectra of compounds **1** and **2**.

File 2 NMR chemical shift calculation for compound **1**.

## References

[1] Blunt J W, Carroll A R, Copp B R, Davis R A, Keyzers R A, Prinsep M R (2018). Nat Prod Rep.

[2] Carroll A R, Copp B R, Davis R A, Keyzers R A, Prinsep M R (2019). Nat Prod Rep.

[3] Hunt B, Vincent A C J (2006). Ambio.

[4] Bell J J (2008). Estuarine, Coastal Shelf Sci.

[5] Indraningrat A A G, Smidt H, Sipkema D (2016). Mar Drugs.

[6] Tian R-M, Sun J, Cai L, Zhang W-P, Zhou G-W, Qiu J-W, Qian P-Y (2016). Environ Microbiol.

[7] Kiran G S, Sekar S, Ramasamy P, Thinesh T, Hassan S, Lipton A N, Ninawe A S, Selvin J (2018). Mar Environ Res.

[8] Blockley A, Elliott D R, Roberts A P, Sweet M (2017). Diversity.

[9] Romano G, Costantini M, Sansone C, Lauritano C, Ruocco N, Ianora A (2017). Mar Environ Res.

[10] Zhang J, Yuan B, Liu D, Gao S, Proksch P, Lin W (2018). Front Chem (Lausanne, Switz).

[11] Kumla D, Dethoup T, Gales L, Pereira J A, Freitas-Silva J, Costa P M, Silva A M S, Pinto M M M, Kijjoa A (2019). Molecules.

[12] Mehbub M F, Lei J, Franco C, Zhang W (2014). Mar Drugs.

[13] Hanif N, Murni A, Tanaka C, Tanaka J (2019). Mar Drugs.

[14] Lin W, Brauers G, Ebel R, Wray V, Berg A, Sudarsono, Proksch P (2003). J Nat Prod.

[15] Rotinsulu H, Yamazaki H, Sugai S, Iwakura N, Wewengkang D S, Sumilat D A, Namikoshi M (2018). J Nat Med.

[16] Sabdaningsih A, Cristianawati O, Sibero M T, Nuryadi H, Radjasa O K, Sabdono A, Trianto A (2017). IOP Conf Ser Earth Environ Sci.

[17] Sibero M T, Radjasa O K, Sabdono A, Trianto A, Triningsih D W, Hutagaol I D (2018). J Appl Pharm Sci.

[18] Sibero M T, Herdikiawan D, Radjasa O K, Sabdono A, Trianto A, Triningsih D W (2018). AACL Bioflux.

[19] Betina V, Sedmera P, Vokoun J, Podojil M (1986). Experientia.

[20] Khokhar S, Pierens G K, Hooper J N A, Ekins M G, Feng Y, Davis R A (2016). J Nat Prod.

[21] Li S, Shao M-W, Lu Y-H, Kong L-C, Jiang D-H, Zhang Y-L (2014). J Agric Food Chem.

[22] Bashyal B P, Gunatilaka A A L (2010). Nat Prod Res.

[23] Nozoe S, Goi M, Morisaki N (1970). Tetrahedron Lett.

[24] Stodůlková E, Kolařík M, Křesinová Z, Kuzma M, Šulc M, Man P, Novák P, Maršík P, Landa P, Olšovská J (2009). Folia Microbiol.

[25] Grimblat N, Zanardi M M, Sarotti A M (2015). J Org Chem.

[26] Fouillaud M, Venkatachalam M, Girard-Valenciennes E, Caro Y, Dufossé L (2016). Mar Drugs.

[27] Cao Q-X, Wei J-H, Deng R, Feng G-K, Zhu X-F, Lan W-J, Li H-J (2017). Chem Biodiversity.

[28] Liu S-Z, Yan X, Tang X-X, Lin J-G, Qiu Y-K (2018). Mar Drugs.

[29] Veitch N C, Simmonds M S J, Blaney W M, Reynolds T (1994). Phytochemistry.

[30] Kjer J, Debbab A, Aly A H, Proksch P (2010). Nat Protoc.

[31] Kim Y, Ogura H, Akasaka K, Oikawa T, Matsuura N, Imada C, Yasuda H, Igarashi Y (2014). Mar Drugs.

[32] (2018). MacroModel.

[33] (2018). Maestro.

[34] (2016). Gaussian 16.

