# Supplementary material for: Two new aromatic polyketides from a sponge-derived Fusarium
Source: Beilstein J Org Chem. 2019 Dec 9;15:2941–7. doi: 10.3762/bjoc.15.289 (PMC6941419; doi:10.3762/bjoc.15.289)

BEILSTEIN JOURNAL OF ORGANIC CHEMISTRY

Supporting Information

for

Two new aromatic polyketides from a sponge-derived *Fusarium*

Mada Triandala Sibero, Tao Zhou, Keisuke Fukaya, Daisuke Urabe,
Ocky K. Karna Radjasa, Agus Sabdono, Agus Trianto and Yasuhiro Igarashi

Beilstein J. Org. Chem. **2019,** *15,* 2941–2947. doi:10.3762/bjoc.15.289

Copies of UV, IR, and NMR spectra of compounds 1 and 2

License and Terms: This is a supporting information file under the terms of the Creative Commons Attribution License (http://creativecommons.org/licenses/by/4.0). Please note that the reuse, redistribution and reproduction in particular requires that the authors and source are credited.
The license is subject to the *Beilstein Journal of Organic Chemistry* terms and conditions: (https://www.beilstein-journals.org/bjoc)

Table of contents

Figure S1. UV spectrum of karimunone A (**1**)
Figure S2. IR spectrum of **1**
Figure S3. ^{1}H NMR spectrum of **1** (500 MHz, DMSO-d_6)
Figure S4. ^{13}C NMR spectrum of **1** (125 MHz, DMSO-d_6)
Figure S5. HSQC spectrum of **1** (500 MHz, DMSO-d_6)
Figure S6. HMBC spectrum of **1** (500 MHz, DMSO-d_6)
Figure S7. UV spectrum of karimunone B (**2**)
Figure S8. IR spectrum of **2**
Figure S9. ^{1}H NMR spectrum of **2** (500 MHz, $CDCl_3$)
Figure S10. ^{13}C NMR spectrum of **2** (125 MHz, $CDCl_3$)
Figure S11. HSQC spectrum of **2** (500 MHz, $CDCl_3$)
Figure S12. HMBC spectrum of **2** (500 MHz, $CDCl_3$)

Figure S1. UV spectrum of karimunone A (**1**)

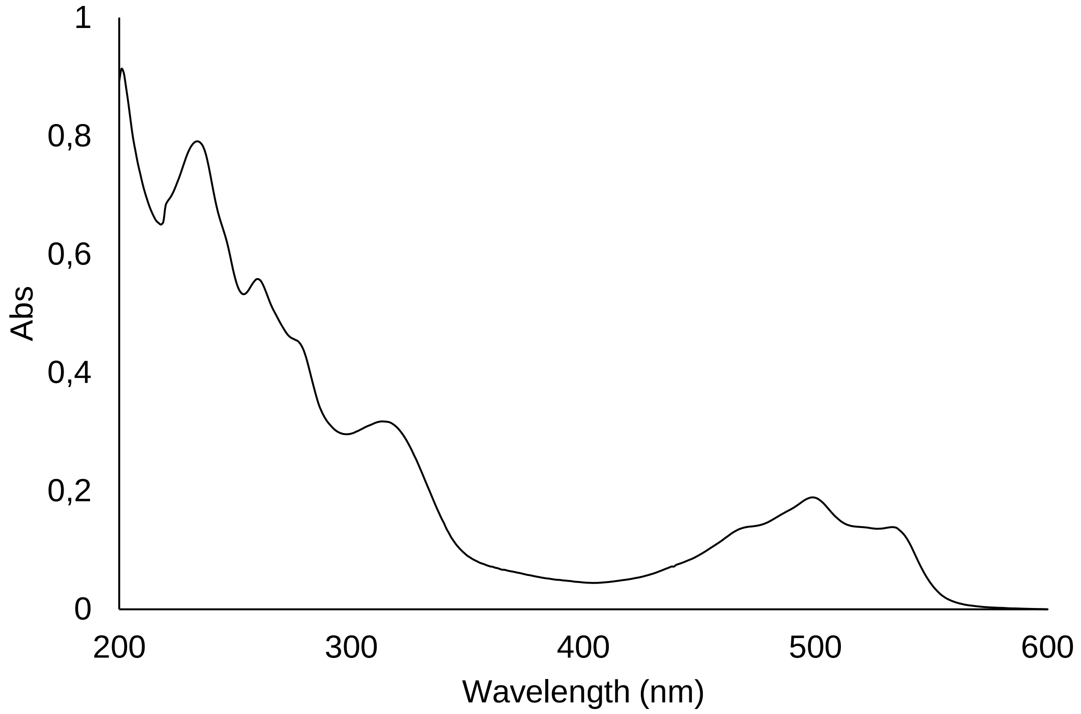

Figure S2. IR spectrum of **1**

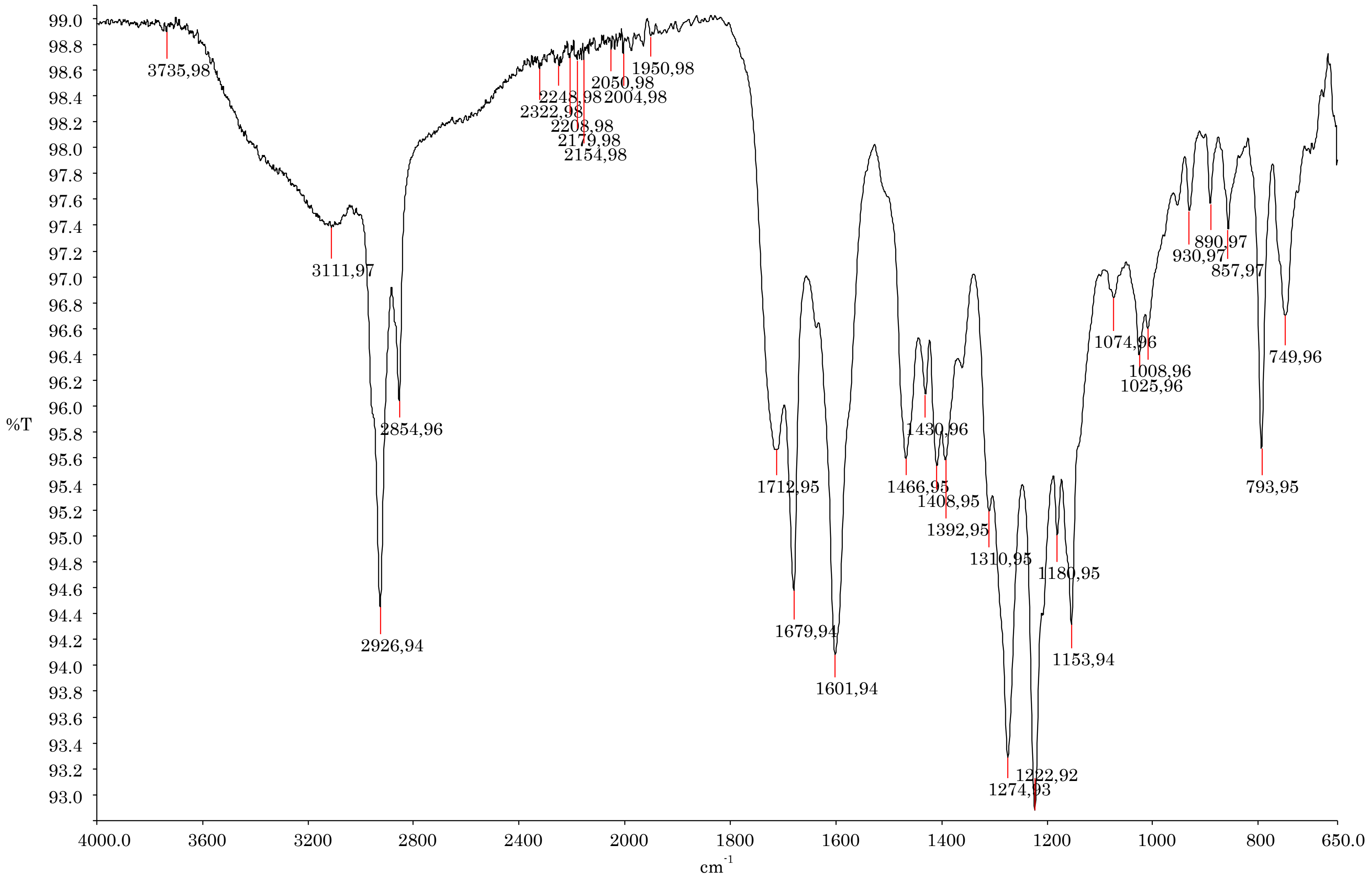

Figure S3. ^{1}H NMR spectrum of **1** (500 MHz, DMSO-d_6)

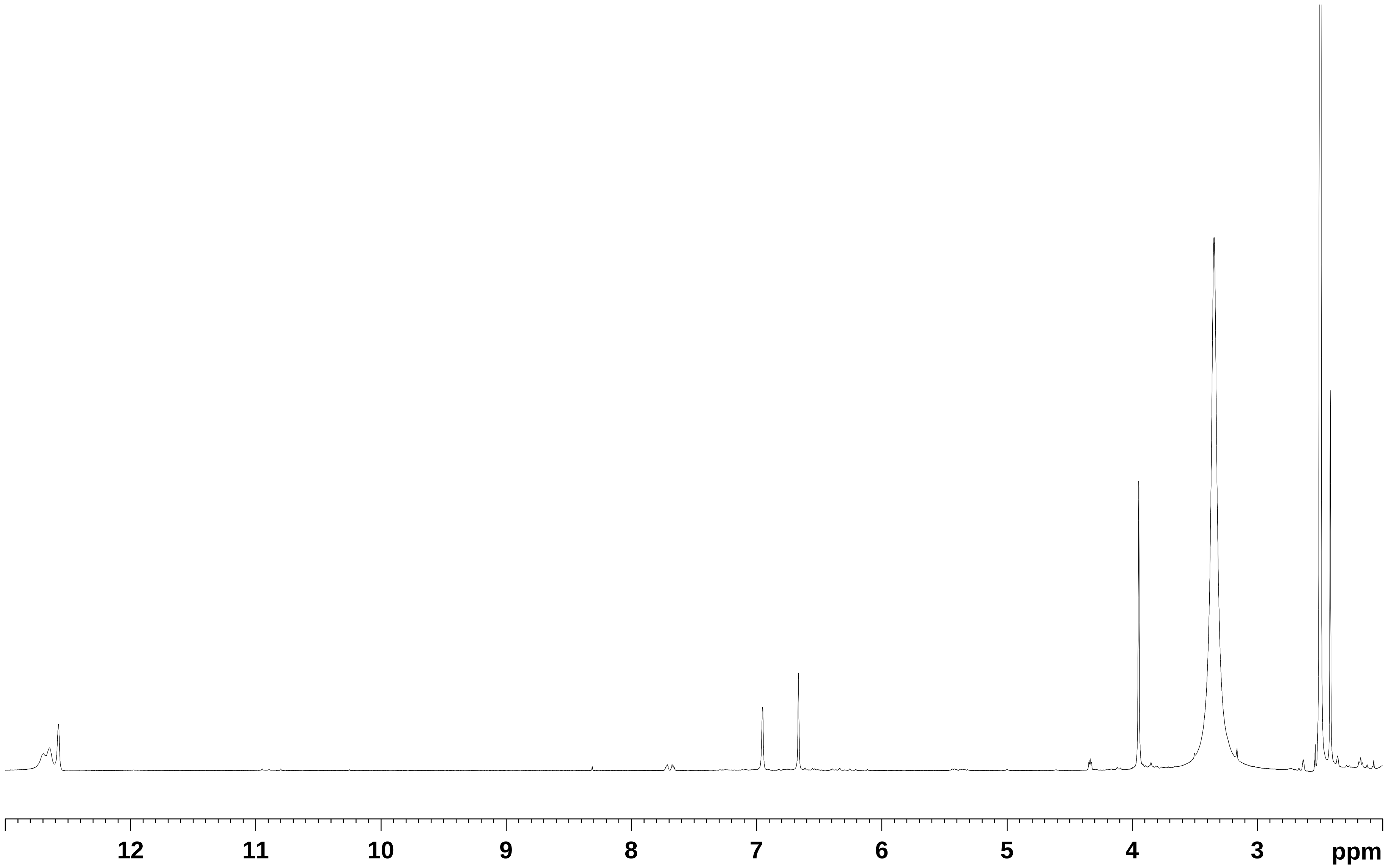

Figure S4. ^{13}C NMR spectrum of **1** (125 MHz, DMSO-d_6)

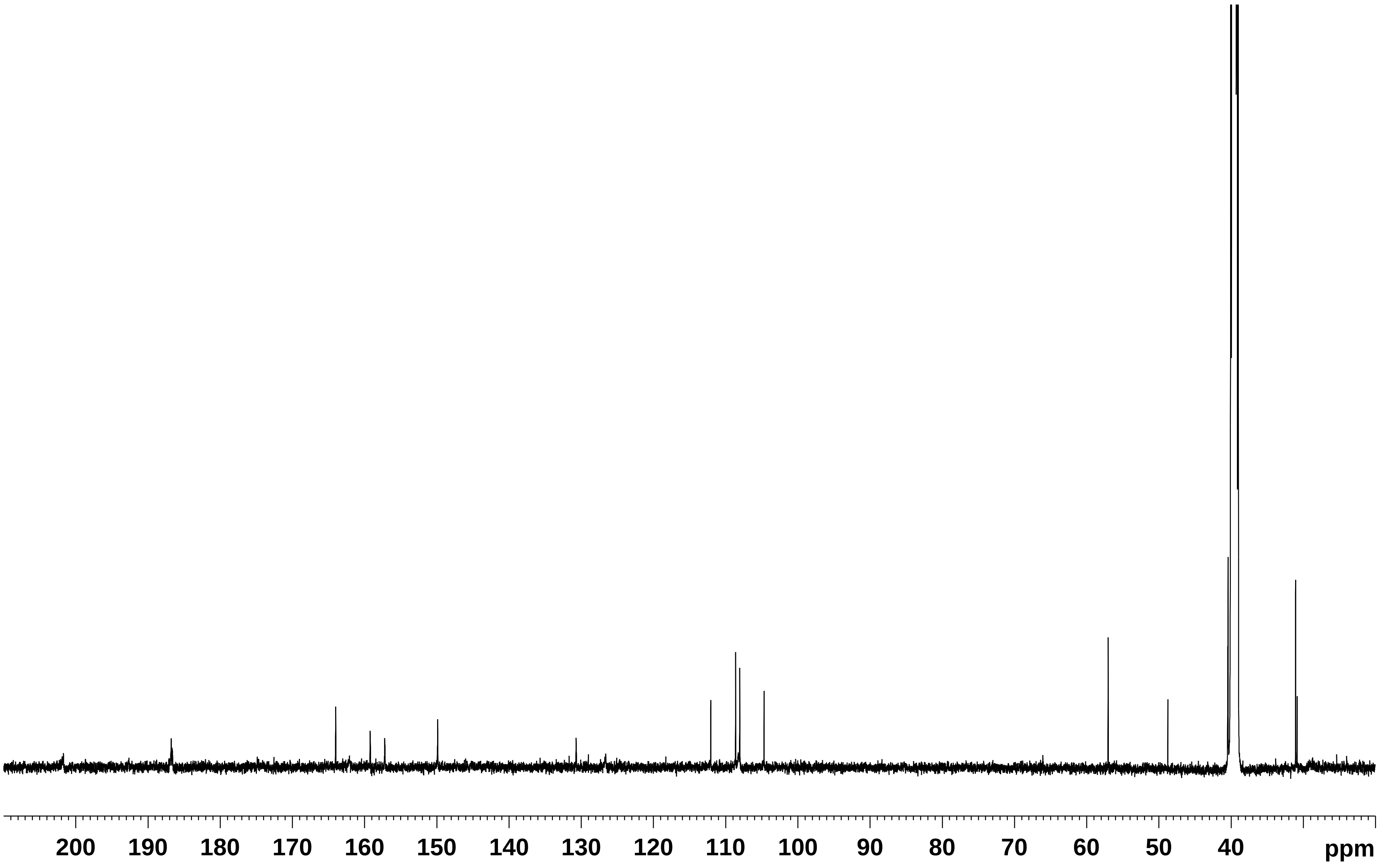

Figure S5. HSQC spectrum of **1** (500 MHz, DMSO-d_6)

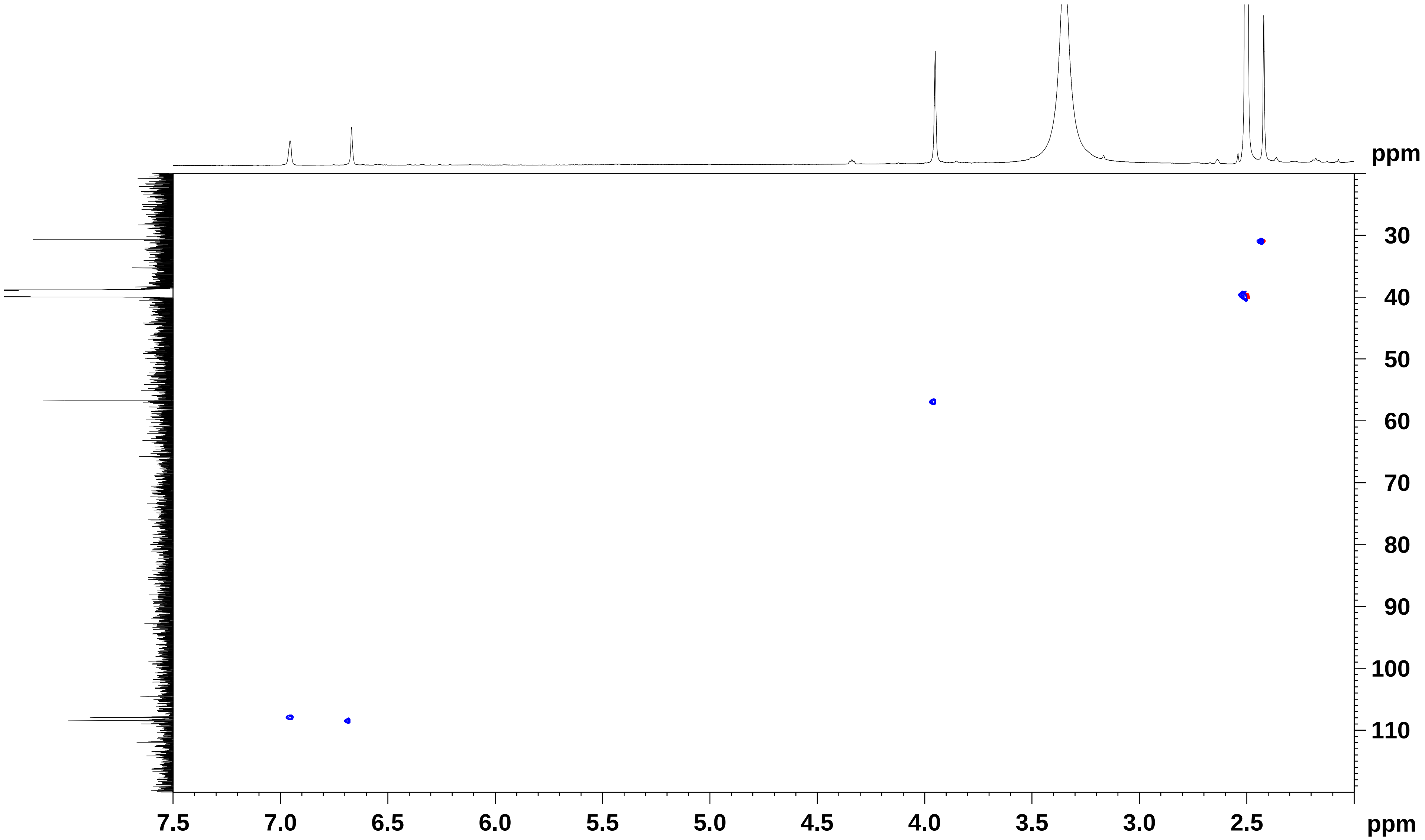

Figure S6. HMBC spectrum of **1** (500 MHz, DMSO-d_6)

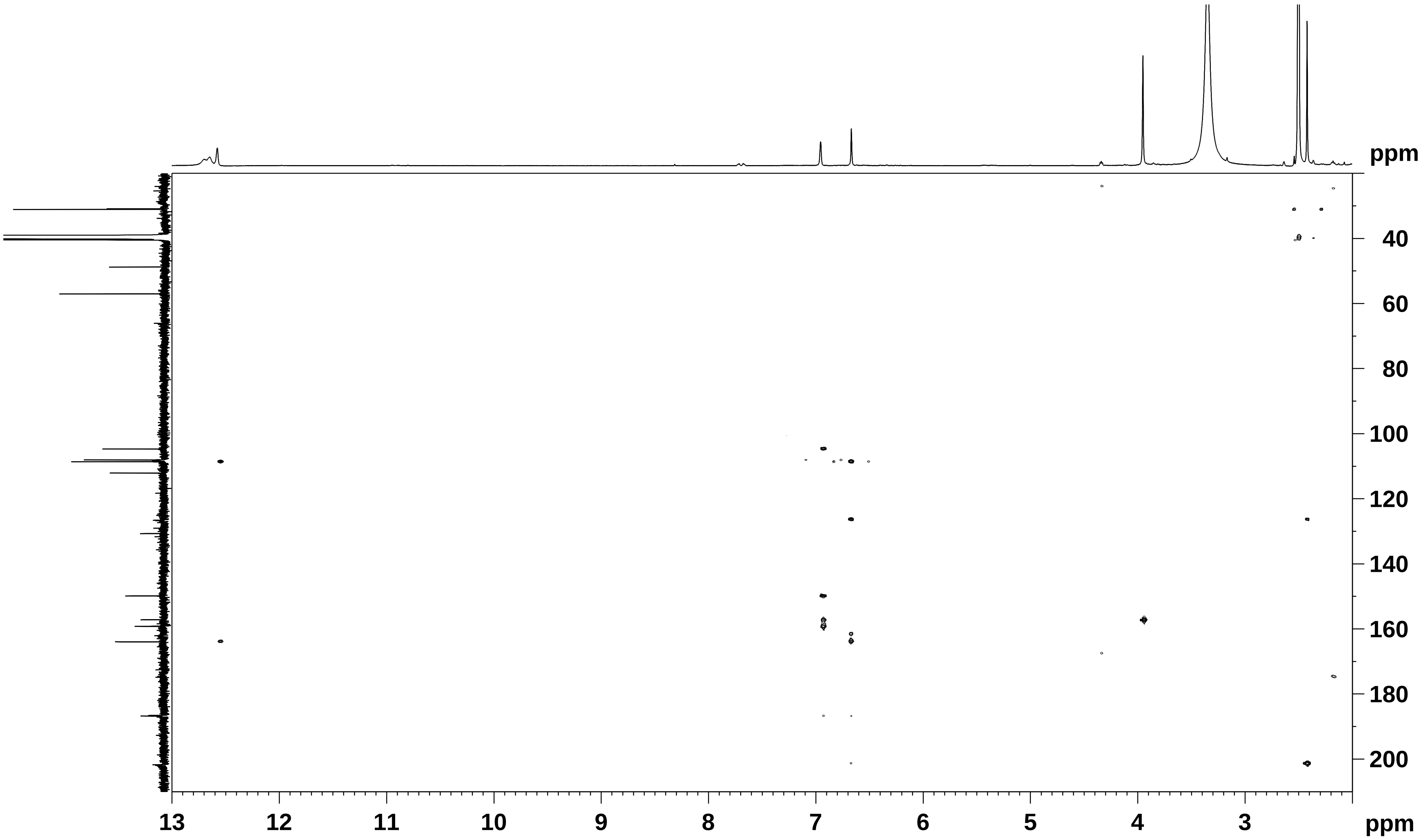

Figure S7. UV spectrum of karimunone B (**2**)

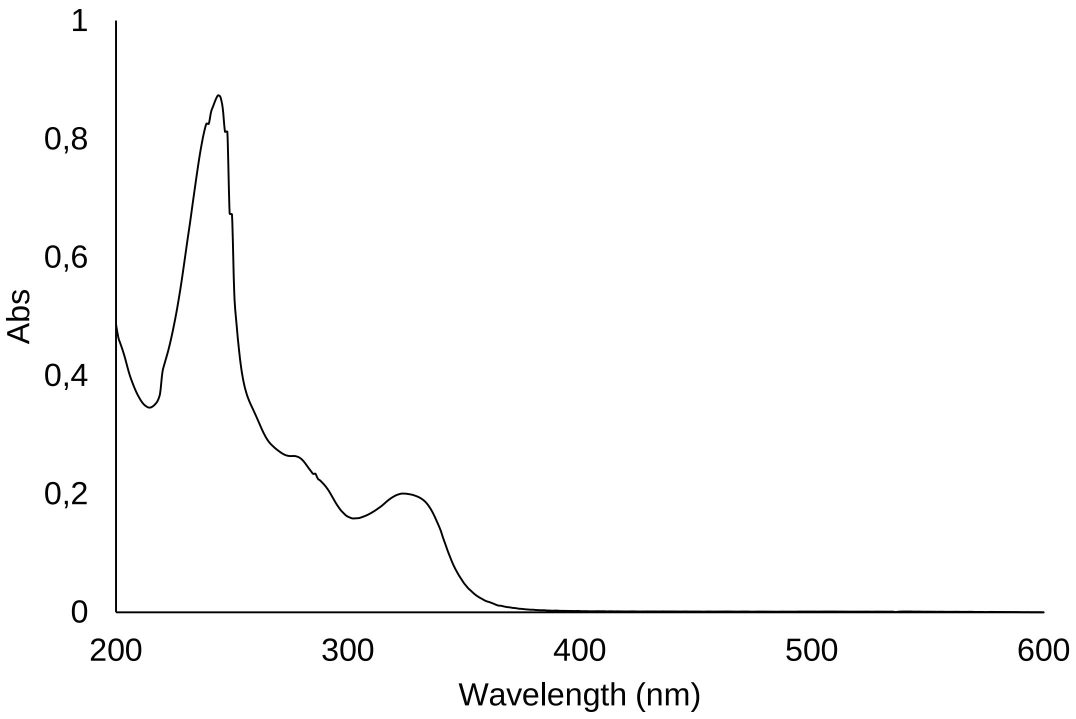

Figure S8. IR spectrum of **2**

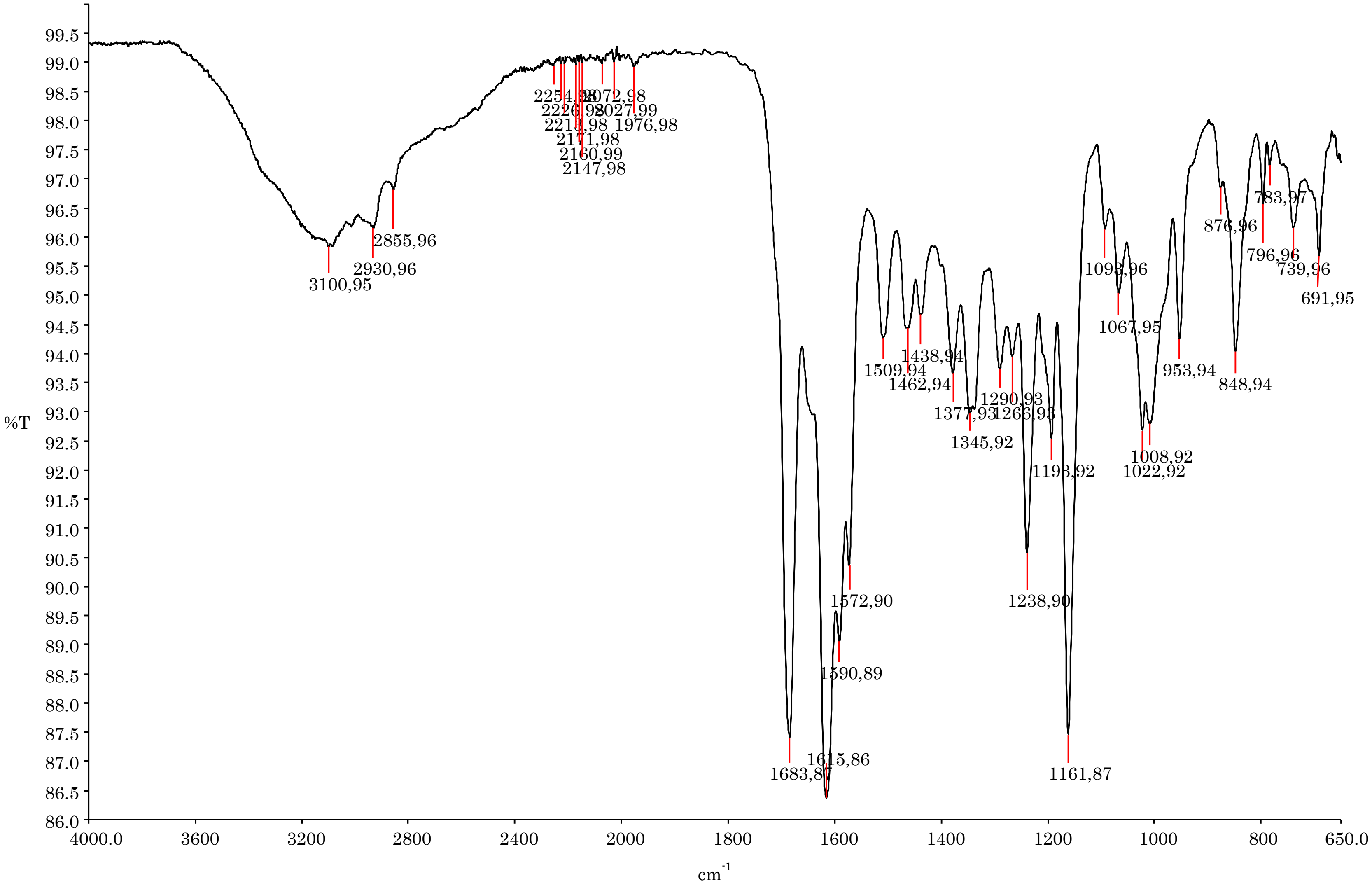

Figure S9. ^{1}H NMR spectrum of **2** (500 MHz, $CDCl_3$)

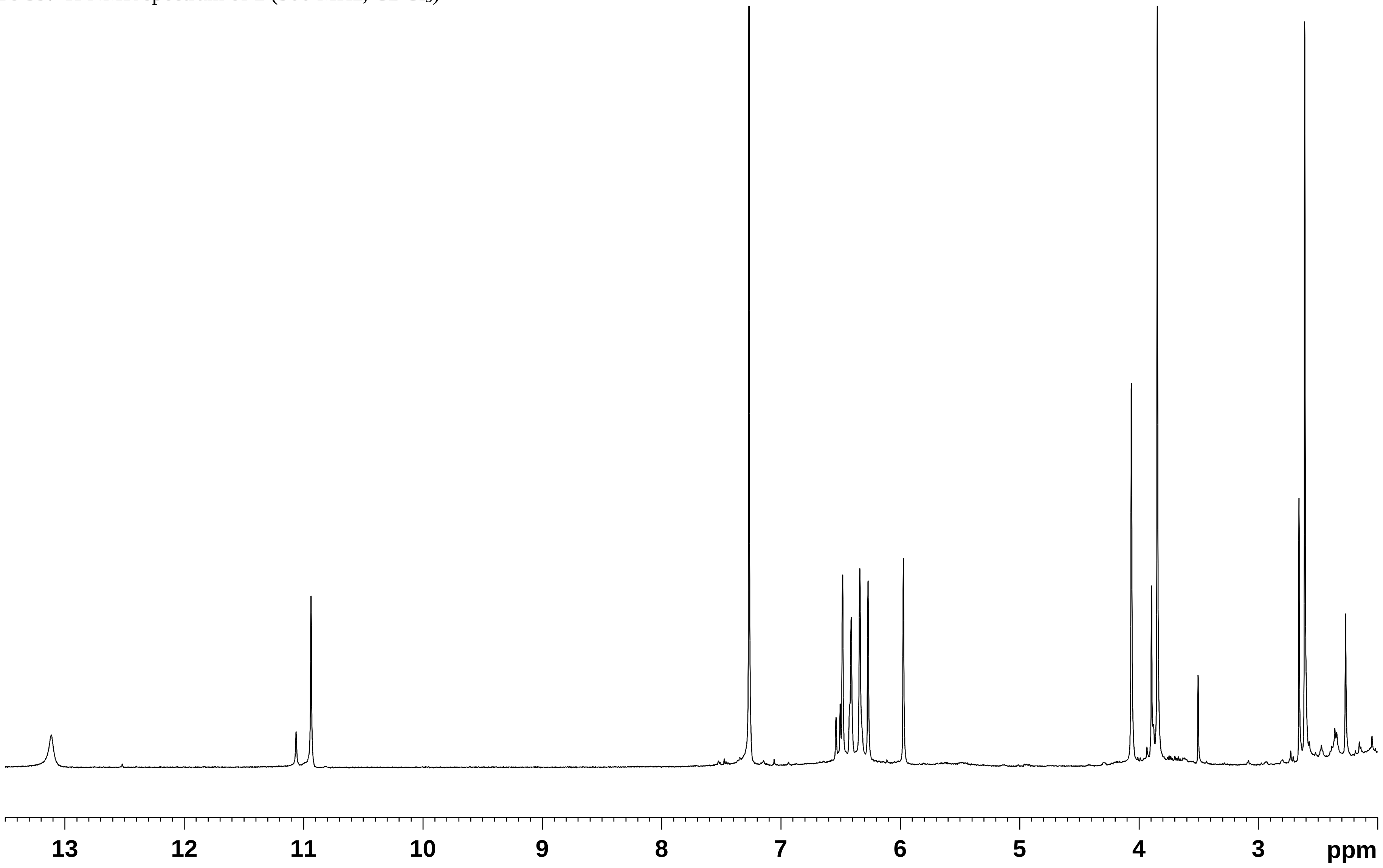

Figure S10. ^{13}C NMR spectrum of **2** (125 MHz, $CDCl_3$)

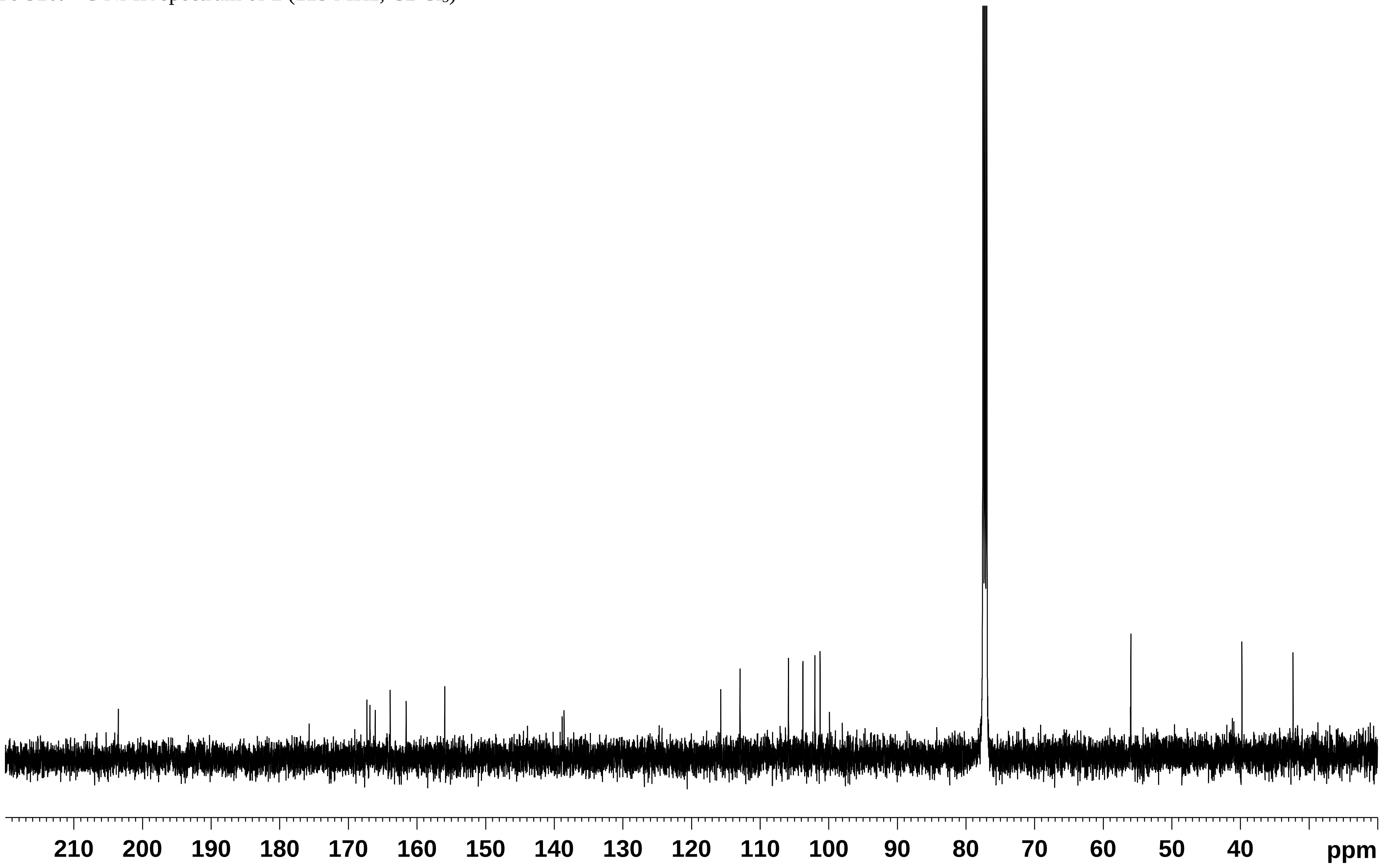

Figure S11. HSQC spectrum of **2** (500 MHz, $CDCl_3$)

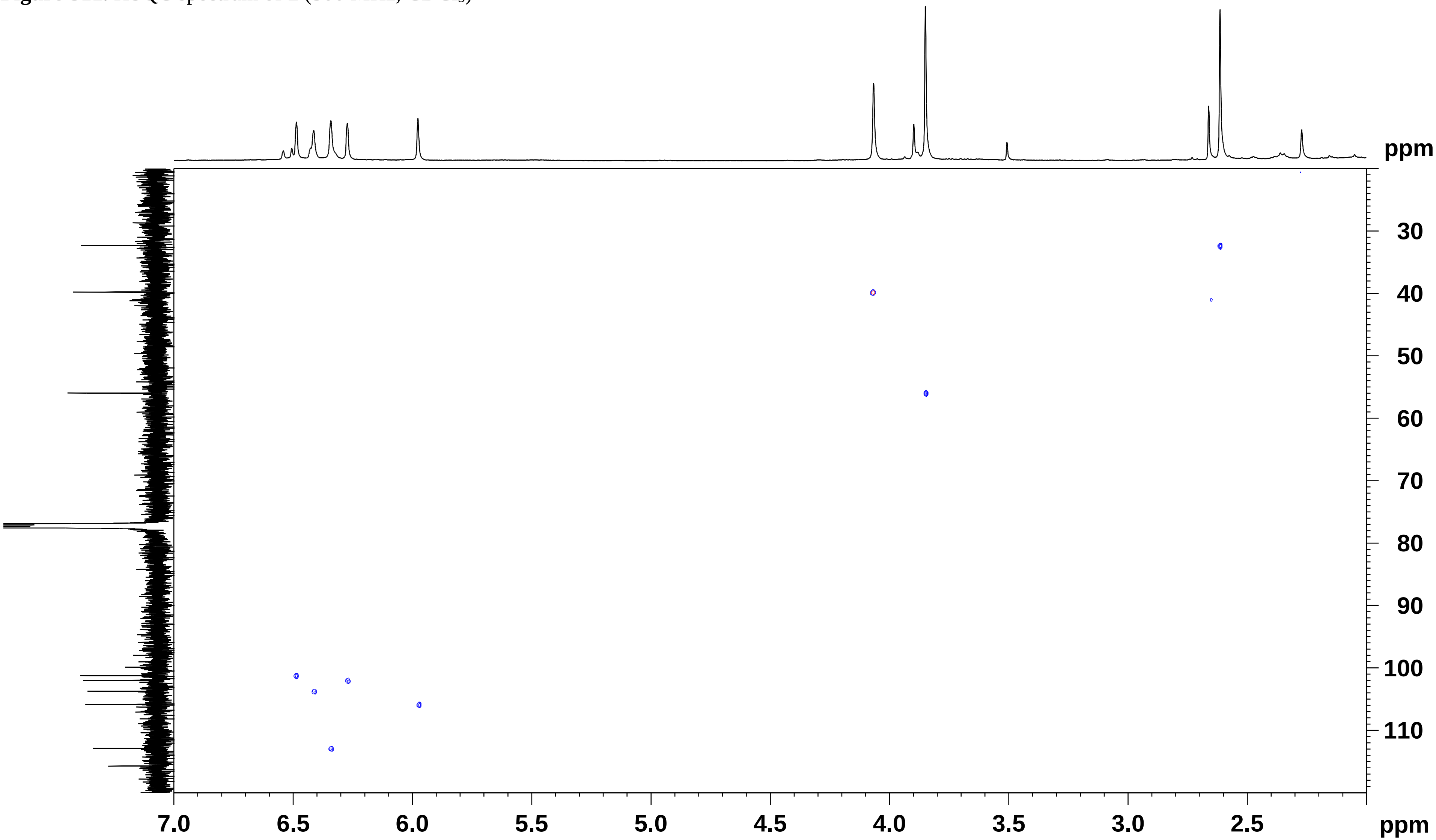

Figure S12. HMBC spectrum of **2** (500 MHz, $CDCl_3$)

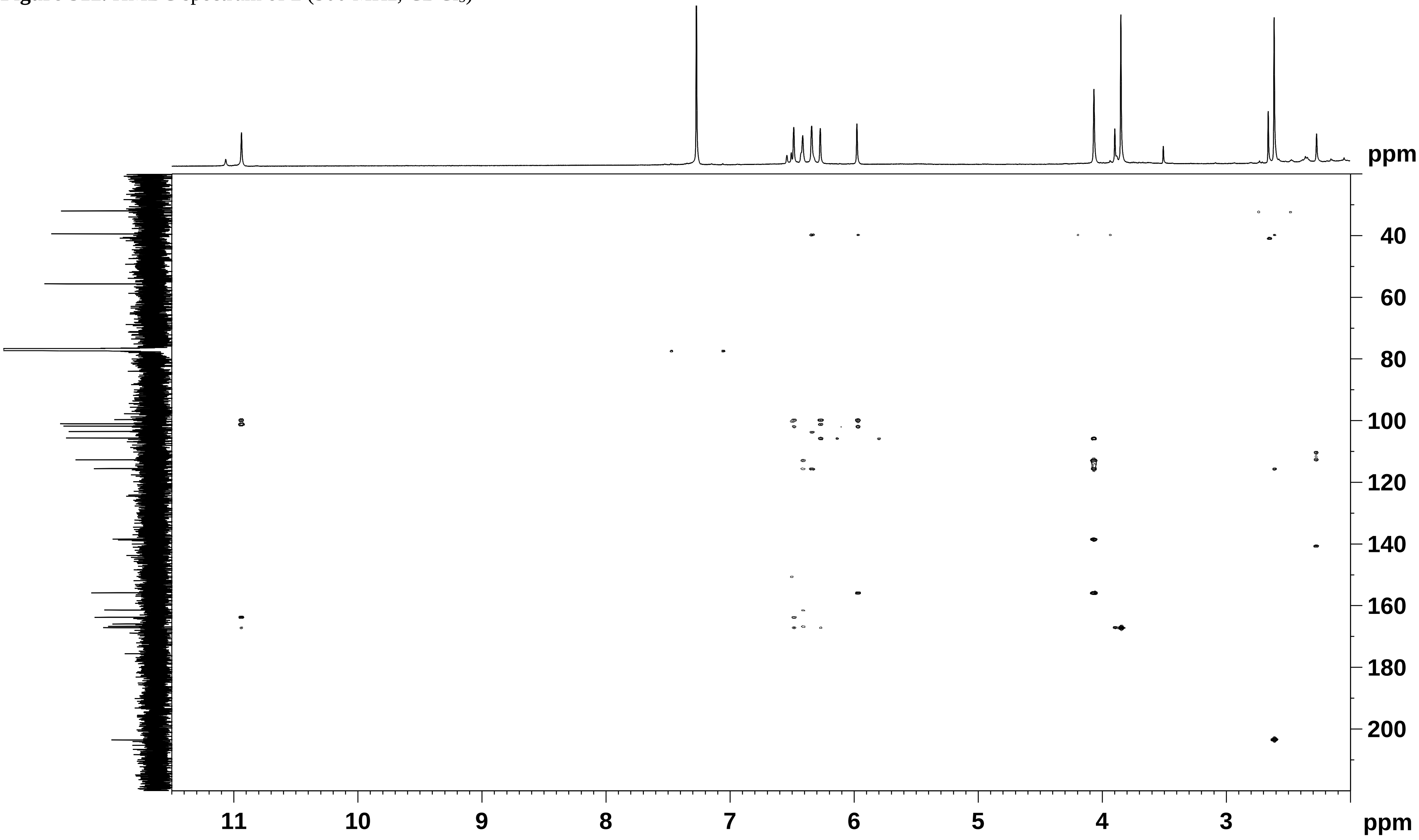

Supplement: File 1 — Copies of UV, IR, and NMR spectra of compounds 1 and 2. [file Beilstein_J_Org_Chem-15-2941-s001.pdf]
