# Supplementary material for: Two new aromatic polyketides from a sponge-derived Fusarium
Source: Beilstein J Org Chem. 2019 Dec 9;15:2941–7. doi: 10.3762/bjoc.15.289 (PMC6941419; doi:10.3762/bjoc.15.289)
Supplement: File 2 — NMR chemical shift calculation for compound 1. [file Beilstein_J_Org_Chem-15-2941-s002.pdf]

BEILSTEIN JOURNAL OF ORGANIC CHEMISTRY

Supporting Information

for

Two new aromatic polyketides from a sponge-derived *Fusarium*

Mada Triandala Sibero, Tao Zhou, Keisuke Fukaya, Daisuke Urabe, Ocky K. Karna Radjasa, Agus Sabdono, Agus Trianto and Yasuhiro Igarashi

Beilstein J. Org. Chem. **2019,** *15,* 2941–2947. doi:10.3762/bjoc.15.289

NMR chemical shift calculation for compound 1

License and Terms: This is a supporting information file under the terms of the Creative Commons Attribution License (http://creativecommons.org/licenses/by/4.0). Please note that the reuse, redistribution and reproduction in particular requires that the authors and source are credited.
The license is subject to the *Beilstein Journal of Organic Chemistry* terms and conditions: (https://www.beilstein-journals.org/bjoc)

Table of contents

	page
• Cartesian coordinates of the optimized structures of **a** and **b**	S2
• Calculated NMR chemical shifts for each conformer of **a** and **b**	S10

Cartesian coordinates of the optimized structures of a and b

Structure, energy and Cartesian coordinate of **a-conformer 1**:

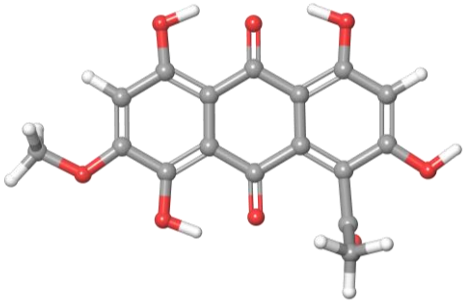

a-conformer 1 (Δ*G* = 0.0 kcal/mol, Boltzmann pop. (298 K) = 69%)

M06-2X/6-311+G(d,p)-PCM(DMSO)//M06-2X/6-31G(d)-PCM(DMSO):		
Gibbs Free Energy (a.u.)	=	−1256.5372187
M06-2X/6-311+G(d,p)-PCM(DMSO): *Electronic energy* (a.u.)	=	−1256.7588507
M06-2X/6-31G(d)-PCM(DMSO): *Zero-point correction* (a.u.)	=	0.270808
Thermal correction to Energy (a.u.)	=	0.291983
Thermal correction to Enthalpy (a.u.)	=	0.292927
Thermal correction to Gibbs Free Energy (a.u.)	=	0.221632

C	2.26967	−1.23710	0.01793
C	1.16475	−0.39446	0.01155
C	1.32851	1.02632	−0.00668
C	2.62161	1.55869	−0.01594
C	3.74509	0.70255	−0.00540
C	3.58578	−0.66363	0.01131
C	−0.17526	−0.98307	0.02482
C	−1.35663	−0.07778	0.03095
C	−1.16860	1.32330	−0.00115
C	0.17300	1.90527	−0.01759
C	−2.62284	−0.64088	0.06044
C	−3.74052	0.22032	0.04692
C	−3.58783	1.59600	0.00791
C	−2.30812	2.15901	−0.01033
O	0.31846	3.15309	−0.04043
O	−0.34834	−2.20715	0.02782
O	−2.23175	3.49162	−0.03950
O	−4.94790	−0.37979	0.06057
C	−2.91452	−2.12956	0.13250
O	−3.15413	−2.64916	1.19964
C	−3.02948	−2.84604	−1.18566
O	2.21109	−2.57071	0.03157
O	2.88072	2.86996	−0.03450
O	4.57948	−1.55955	0.02182
C	5.91238	−1.06205	0.01738
H	4.72478	1.16366	−0.01178
H	−4.44837	2.25736	−0.00561
H	−1.27403	3.73267	−0.04828
H	−5.65613	0.28343	0.03290
H	−2.17281	−2.61841	−1.82399
H	−3.11031	−3.92166	−1.02191
H	−3.93299	−2.48470	−1.69103
H	1.25645	−2.81778	0.03247
H	2.01300	3.34022	−0.04179
H	6.55649	−1.93902	0.02830
H	6.10138	−0.47229	−0.88485
H	6.09947	−0.45136	0.90597

Structure, energy and Cartesian coordinate of **a-conformer 2**:

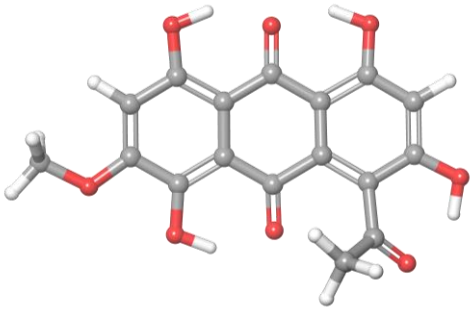

a-conformer 2 (ΔG = 0.5 kcal/mol, Boltzmann pop. (298 K) = 31%)

M06-2X/6-311+G(d,p)-PCM(DMSO)//M06-2X/6-31G(d)-PCM(DMSO):			
	Gibbs Free Energy (a.u.)	=	−1256.5364662
M06-2X/6-311+G(d,p)-PCM(DMSO):	*Electronic energy* (a.u.)	=	−1256.7598442
M06-2X/6-31G(d)-PCM(DMSO):	*Zero-point correction* (a.u.)	=	0.271800
	Thermal correction to Energy (a.u.)	=	0.292435
	Thermal correction to Enthalpy (a.u.)	=	0.293379
	Thermal correction to Gibbs Free Energy (a.u.)	=	0.223378

C	−2.26776	−1.21192	−0.18932
C	−1.15757	−0.37982	−0.12579
C	−1.30945	1.03267	0.02696
C	−2.59636	1.56872	0.13521
C	−3.72518	0.71997	0.10710
C	−3.57740	−0.63884	−0.05018
C	0.17786	−0.96535	−0.24599
C	1.36890	−0.08828	−0.04666
C	1.19178	1.30549	−0.03743
C	−0.14697	1.89921	0.03603
C	2.63904	−0.67255	−0.02986
C	3.76519	0.18561	−0.15795
C	3.60963	1.56725	−0.13308
C	2.34478	2.13729	−0.04039
O	−0.27676	3.14473	0.12321
O	0.34200	−2.15381	−0.53852
O	2.27114	3.46806	0.00012
O	5.01208	−0.28400	−0.27506
C	2.91444	−2.11754	0.23779
O	3.85990	−2.66259	−0.32205
C	2.20543	−2.84128	1.35735
O	−2.22046	−2.53458	−0.36363
O	−2.84354	2.87552	0.26484
O	−4.57701	−1.52609	−0.09939
C	−5.90423	−1.02801	0.02404
H	−4.69998	1.18177	0.20128
H	4.48265	2.20658	−0.19412
H	1.31624	3.71318	0.05771
H	4.96312	−1.25195	−0.42939
H	1.38484	−2.27421	1.79283
H	1.84104	−3.80479	0.99653
H	2.95765	−3.02997	2.13018
H	−1.27414	−2.77684	−0.49142
H	−1.97495	3.34277	0.25334
H	−6.55459	−1.89815	−0.03805
H	−6.04087	−0.52864	0.98813
H	−6.13244	−0.33339	−0.79011

Structure, energy and Cartesian coordinate of **a-conformer 3**:

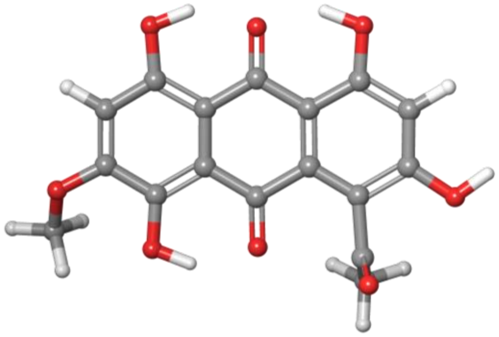

a-conformer 3 (ΔG = 3.3 kcal/mol, Boltzmann pop. (298 K) = 0.3%)

M06-2X/6-311+G(d,p)-PCM(DMSO)//M06-2X/6-31G(d)-PCM(DMSO):		
Gibbs Free Energy (a.u.)	=	−1256.5319414
M06-2X/6-311+G(d,p)-PCM(DMSO): *Electronic energy* (a.u.)	=	−1256.7519884
M06-2X/6-31G(d)-PCM(DMSO): *Zero-point correction* (a.u.)	=	0.269972
Thermal correction to Energy (a.u.)	=	0.291470
Thermal correction to Enthalpy (a.u.)	=	0.292414
Thermal correction to Gibbs Free Energy (a.u.)	=	0.220047

C	−2.40509	−0.99559	0.15732
C	−1.24150	−0.22816	0.09695
C	−1.30558	1.19619	0.05067
C	−2.55726	1.82331	0.06810
C	−3.72799	1.04043	0.10805
C	−3.67133	−0.33274	0.14036
C	0.05684	−0.90735	0.11032
C	1.29904	−0.09006	0.04806
C	1.20973	1.31932	−0.02016
C	−0.08659	1.99274	−0.01178
C	2.52188	−0.74120	0.05293
C	3.69710	0.03731	−0.02642
C	3.64105	1.41874	−0.10014
C	2.40413	2.07113	−0.09124
O	−0.14795	3.24458	−0.05797
O	0.14526	−2.13896	0.16345
O	2.42184	3.40415	−0.15590
O	4.85676	−0.64933	−0.03880
C	2.71180	−2.24429	0.16195
O	2.96097	−2.74633	1.23515
C	2.71922	−3.00709	−1.13500
O	−2.42165	−2.32980	0.25260
O	−2.72631	3.15014	0.03199
O	−4.83218	−1.01587	0.22553
C	−5.03130	−2.13868	−0.64462
H	−4.69392	1.53283	0.10361
H	4.54439	2.01722	−0.16179
H	1.48561	3.71569	−0.13686
H	5.61109	−0.04309	−0.11424
H	2.73306	−4.08009	−0.93817
H	1.85338	−2.74010	−1.74513
H	3.62261	−2.72591	−1.68914
H	−1.48377	−2.63643	0.24229
H	−1.83130	3.56066	−0.00978
H	−6.10442	−2.17869	−0.83211
H	−4.49983	−1.99442	−1.58885
H	−4.69577	−3.06033	−0.17038

Structure, energy and Cartesian coordinate of **a-conformer 4**:

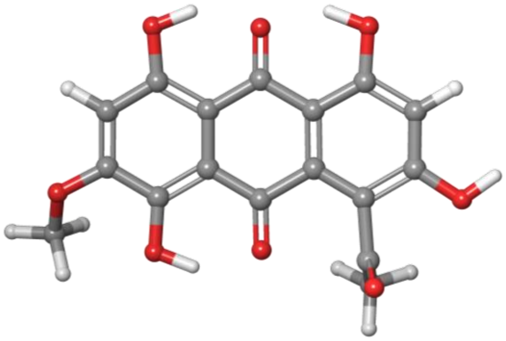

a-conformer 4 (ΔG = 3.4 kcal/mol, Boltzmann pop. (298 K) = 0.2%)

M06-2X/6-311+G(d,p)-PCM(DMSO)//M06-2X/6-31G(d)-PCM(DMSO):		
Gibbs Free Energy (a.u.)	=	−1256.5318302
M06-2X/6-311+G(d,p)-PCM(DMSO): *Electronic energy* (a.u.)	=	−1256.7520482
M06-2X/6-31G(d)-PCM(DMSO): *Zero-point correction* (a.u.)	=	0.270120
Thermal correction to Energy (a.u.)	=	0.291571
Thermal correction to Enthalpy (a.u.)	=	0.292515
Thermal correction to Gibbs Free Energy (a.u.)	=	0.220218

C	2.40496	−1.00108	−0.12361
C	1.24222	−0.23174	−0.07265
C	1.30720	1.19327	−0.06289
C	2.55907	1.81897	−0.10375
C	3.72925	1.03462	−0.12737
C	3.67167	−0.33890	−0.12545
C	−0.05642	−0.91029	−0.05738
C	−1.29686	−0.09135	0.01726
C	−1.20750	1.31963	0.02330
C	0.08879	1.99195	−0.01843
C	−2.51880	−0.74214	0.06749
C	−3.69374	0.03900	0.11677
C	−3.63821	1.42243	0.11794
C	−2.40140	2.07379	0.07634
O	0.15059	3.24463	−0.01641
O	−0.14668	−2.14199	−0.10398
O	−2.41891	3.40838	0.08614
O	−4.85366	−0.64667	0.14871
C	−2.70540	−2.24929	0.09979
O	−2.86031	−2.81836	1.15719
C	−2.82829	−2.92822	−1.23725
O	2.42016	−2.33700	−0.19112
O	2.72880	3.14620	−0.10505
O	4.83169	−1.02487	−0.19738
C	5.03142	−2.12722	0.69846
H	4.69545	1.52637	−0.13918
H	−4.54150	2.02306	0.15397
H	−1.48278	3.71874	0.05051
H	−5.60959	−0.03785	0.15991
H	−2.83489	−4.01156	−1.10852
H	−3.77156	−2.60872	−1.69587
H	−2.01304	−2.62543	−1.89831
H	1.48187	−2.64228	−0.17646
H	1.83401	3.55815	−0.07366
H	6.10462	−2.16280	0.88645
H	4.50025	−1.96138	1.63928
H	4.69581	−3.05956	0.24568

Structure, energy and Cartesian coordinate of **b-conformer 1**:

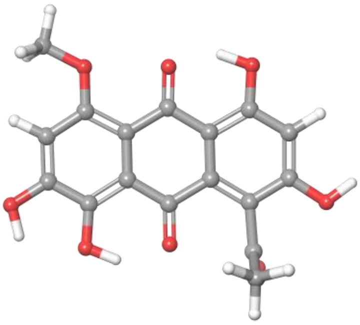

b-conformer 1 (ΔG = 0.0 kcal/mol, Boltzmann pop. (298 K) = 73%)

M06-2X/6-311+G(d,p)-PCM(DMSO)//M06-2X/6-31G(d)-PCM(DMSO):			
	Gibbs Free Energy (a.u.)	=	−1256.5263727
M06-2X/6-311+G(d,p)-PCM(DMSO):	*Electronic energy* (a.u.)	=	−1256.7470117
M06-2X/6-31G(d)-PCM(DMSO):	*Zero-point correction* (a.u.)	=	0.270485
	Thermal correction to Energy (a.u.)	=	0.291933
	Thermal correction to Enthalpy (a.u.)	=	0.292878
	Thermal correction to Gibbs Free Energy (a.u.)	=	0.220639

C	1.95909	−1.90465	0.01726
C	1.08681	−0.82104	0.01005
C	1.58085	0.51734	−0.00385
C	2.97419	0.72039	−0.00402
C	3.84747	−0.38530	0.00635
C	3.35466	−1.67077	0.01589
C	−0.35607	−1.10582	0.02188
C	−1.31405	0.02456	0.03188
C	−0.81972	1.34369	−0.00261
C	0.62751	1.63918	−0.02268
C	−2.67352	−0.25463	0.06368
C	−3.57665	0.82623	0.04969
C	−3.12467	2.13451	0.00843
C	−1.75238	2.40587	−0.01292
O	1.00316	2.81912	−0.05538
O	−0.78078	−2.26546	0.02110
O	−1.38823	3.68986	−0.04595
O	−4.88754	0.50309	0.06392
C	−3.27698	−1.64565	0.13486
O	−3.61776	−2.10702	1.20148
C	−3.54564	−2.31784	−1.18447
O	1.61006	−3.20355	0.02732
O	3.44499	1.97797	−0.01259
O	4.19601	−2.71783	0.02432
C	4.85110	2.18198	−0.00784
H	4.92208	−0.25898	0.00638
H	−3.81873	2.96911	−0.00572
H	−0.39601	3.69885	−0.05896
H	−5.43524	1.30376	0.03380
H	−3.85936	−3.35019	−1.02289
H	−4.34772	−1.76684	−1.68970
H	−2.65953	−2.28154	−1.82234
H	0.62509	−3.24746	0.02545
H	3.66630	−3.53551	0.03130
H	5.31607	1.74882	−0.89886
H	4.98743	3.26206	−0.01410
H	5.30838	1.76007	0.89250

Structure, energy and Cartesian coordinate of **b-conformer 2**:

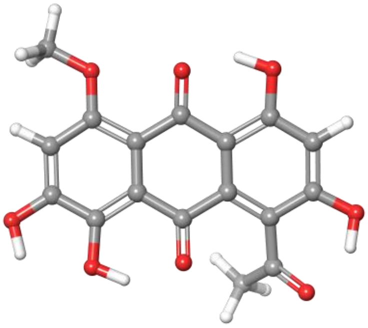

b-conformer 2 (ΔG = 0.6 kcal/mol, Boltzmann pop. (298 K) = 25%)

M06-2X/6-311+G(d,p)-PCM(DMSO)//M06-2X/6-31G(d)-PCM(DMSO):		
Gibbs Free Energy (a.u.)	=	−1256.5260183
M06-2X/6-311+G(d,p)-PCM(DMSO): *Electronic energy* (a.u.)	=	−1256.7485673
M06-2X/6-31G(d)-PCM(DMSO): *Zero-point correction* (a.u.)	=	0.271350
Thermal correction to Energy (a.u.)	=	0.292228
Thermal correction to Enthalpy (a.u.)	=	0.293172
Thermal correction to Gibbs Free Energy (a.u.)	=	0.222549

C	1.97033	−1.86739	−0.23145
C	1.08653	−0.79573	−0.18291
C	1.55951	0.53947	−0.03332
C	2.94393	0.75312	0.10590
C	3.82865	−0.34326	0.09146
C	3.35634	−1.62616	−0.07737
C	−0.35030	−1.08476	−0.29771
C	−1.32710	0.01048	−0.06061
C	−0.85479	1.32765	−0.09026
C	0.59199	1.64595	−0.06677
C	−2.69222	−0.29901	0.00424
C	−3.61558	0.77370	−0.10795
C	−3.16833	2.09067	−0.12754
C	−1.80928	2.38337	−0.09001
O	0.94786	2.83127	−0.05967
O	−0.75490	−2.20635	−0.61157
O	−1.45563	3.66840	−0.09566
O	−4.93804	0.57901	−0.16964
C	−3.24825	−1.64876	0.31415
O	−4.32845	−1.98041	−0.16559
C	−2.62632	−2.51863	1.38120
O	1.64051	−3.16091	−0.40052
O	3.39102	2.00981	0.25519
O	4.20987	−2.66257	−0.10345
C	4.78730	2.22432	0.41112
H	4.89713	−0.21103	0.19959
H	−3.88763	2.89978	−0.18082
H	−0.46385	3.69077	−0.08578
H	−5.09889	−0.38228	−0.28990
H	−2.45945	−3.52173	0.98429
H	−1.69575	−2.12104	1.78228
H	−3.36058	−2.59148	2.18957
H	0.67029	−3.20499	−0.56118
H	3.69616	−3.47905	−0.24018
H	4.90504	3.30108	0.51973
H	5.16596	1.72164	1.30639
H	5.33933	1.88217	−0.46964

Structure, energy and Cartesian coordinate of **b-conformer 3**:

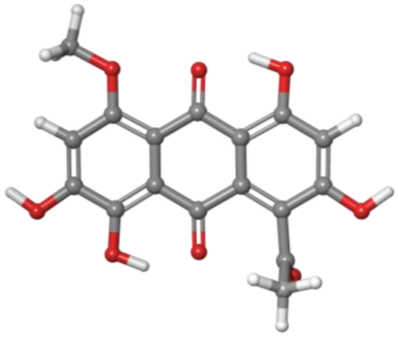

b-conformer 3 (ΔG = 2.5 kcal/mol, Boltzmann pop. (298 K) = 1.1%)

M06-2X/6-311+G(d,p)-PCM(DMSO)//M06-2X/6-31G(d)-PCM(DMSO):		
Gibbs Free Energy (a.u.)	=	−1256.5230671
M06-2X/6-311+G(d,p)-PCM(DMSO): *Electronic energy* (a.u.)	=	−1256.7430241
M06-2X/6-31G(d)-PCM(DMSO): *Zero-point correction* (a.u.)	=	0.270121
Thermal correction to Energy (a.u.)	=	0.291688
Thermal correction to Enthalpy (a.u.)	=	0.292633
Thermal correction to Gibbs Free Energy (a.u.)	=	0.219957

C	1.95682	−1.92304	0.01673
C	1.08582	−0.83198	0.00866
C	1.57479	0.51056	−0.00519
C	2.96415	0.71830	−0.00400
C	3.83743	−0.38880	0.00742
C	3.35520	−1.67878	0.01687
C	−0.35789	−1.10995	0.02015
C	−1.31554	0.02208	0.03110
C	−0.82323	1.34109	−0.00380
C	0.62296	1.63404	−0.02508
C	−2.67524	−0.25716	0.06427
C	−3.57839	0.82359	0.05148
C	−3.12743	2.13243	0.00962
C	−1.75553	2.40376	−0.01336
O	1.00054	2.81364	−0.05941
O	−0.79417	−2.26614	0.01870
O	−1.38994	3.68742	−0.04702
O	−4.88943	0.50048	0.06722
C	−3.28006	−1.64752	0.13547
O	−3.62395	−2.10780	1.20168
C	−3.54961	−2.31969	−1.18388
O	1.58605	−3.20873	0.02623
O	3.43636	1.97550	−0.01208
O	4.13844	−2.77204	0.02692
C	4.84191	2.18201	−0.00454
H	4.91086	−0.24170	0.00808
H	−3.82208	2.96655	−0.00383
H	−0.39759	3.69464	−0.06143
H	−5.43693	1.30129	0.03773
H	−2.66607	−2.27865	−1.82487
H	−3.85812	−3.35365	−1.02234
H	−4.35639	−1.77208	−1.68534
H	0.60079	−3.22510	0.02308
H	5.07296	−2.51060	0.02790
H	5.30984	1.75122	−0.89524
H	4.97606	3.26239	−0.01000
H	5.29870	1.76153	0.89681

Structure, energy and Cartesian coordinate of **b-conformer 4**:

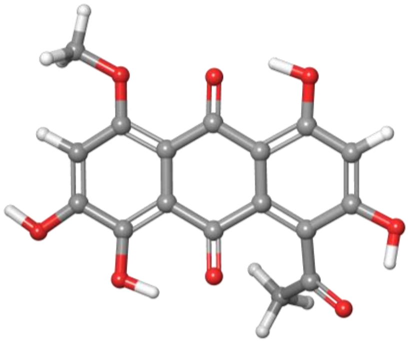

b-conformer 4 (ΔG = 3.0 kcal/mol, Boltzmann pop. (298 K) = 0.5%)

M06-2X/6-311+G(d,p)-PCM(DMSO)//M06-2X/6-31G(d)-PCM(DMSO):			
	Gibbs Free Energy (a.u.)	=	−1256.5222624
M06-2X/6-311+G(d,p)-PCM(DMSO):	*Electronic energy* (a.u.)	=	−1256.7447054
M06-2X/6-31G(d)-PCM(DMSO):	*Zero-point correction* (a.u.)	=	0.271356
	Thermal correction to Energy (a.u.)	=	0.292266
	Thermal correction to Enthalpy (a.u.)	=	0.293210
	Thermal correction to Gibbs Free Energy (a.u.)	=	0.222443

C	1.96751	−1.88359	−0.24247
C	1.08514	−0.80447	−0.19636
C	1.55223	0.53450	−0.04297
C	2.93167	0.75242	0.10543
C	3.81587	−0.34592	0.09906
C	3.35457	−1.63313	−0.07262
C	−0.35227	−1.08641	−0.31471
C	−1.32842	0.00825	−0.06628
C	−0.85957	1.32574	−0.09690
C	0.58603	1.64246	−0.08085
C	−2.69283	−0.30299	0.00811
C	−3.61827	0.76880	−0.09538
C	−3.17348	2.08682	−0.11726
C	−1.81476	2.38088	−0.08978
O	0.94360	2.82755	−0.08102
O	−0.76808	−2.20162	−0.63929
O	−1.46079	3.66589	−0.09780
O	−4.94090	0.57220	−0.14712
C	−3.24442	−1.65376	0.32004
O	−4.32992	−1.98510	−0.14824
C	−2.60988	−2.52683	1.37713
O	1.61669	−3.16327	−0.42124
O	3.37931	2.00880	0.25711
O	4.14938	−2.71710	−0.09989
C	4.77339	2.22493	0.42810
H	4.88203	−0.19335	0.21775
H	−3.89455	2.89470	−0.16470
H	−0.46887	3.68754	−0.09663
H	−5.10072	−0.38950	−0.26673
H	−2.44230	−3.52713	0.97351
H	−1.67726	−2.12763	1.77192
H	−3.33664	−2.60691	2.19154
H	0.64604	−3.17888	−0.58125
H	5.07544	−2.45373	0.02177
H	5.14325	1.72382	1.32802
H	5.33580	1.88426	−0.44673
H	4.88851	3.30188	0.53769

• Calculated NMR chemical shifts for each conformer of **a** and **b**

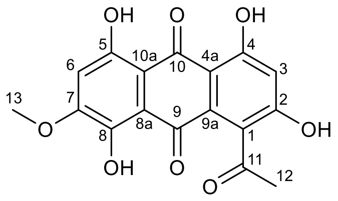

a

**a***			**a-conformer 1** (69%)**		**a-conformer 2** (31%)**		**a-conformer 3** (0.3%)**		**a-conformer 4** (0.2%)**	
position	$\delta_{C(calc)}$	$\delta_{H(calc)}$	$\delta_{C(calc)}$	$\delta_{H(calc)}$	$\delta_{C(calc)}$	$\delta_{H(calc)}$	$\delta_{C(calc)}$	$\delta_{H(calc)}$	$\delta_{C(calc)}$	$\delta_{H(calc)}$
1	122.8		124.9		118.0		125.0		125.1	
2	156.7		155.5		159.4		156.0		156.0	
3	107.5	6.89	107.1	6.85	108.3	7.00	107.1	6.87	107.0	6.87
4	162.3		161.9		163.2		162.0		162.0	
4a	110.2		109.6		111.5		109.4		109.5	
5	157.1		157.0		157.5		155.8		155.8	
6	106.6	6.93	106.4	6.90	106.7	6.99	117.5	7.19	117.5	7.19
7	155.4		155.3		155.7		156.1		156.2	
8	148.3		148.3		148.4		150.6		150.5	
8a	111.9		111.8		112.2		113.4		113.4	
9	183.4		183.0		184.3		183.5		183.5	
9a	133.8		132.3		137.0		132.1		132.2	
10	182.8		182.8		182.8		183.3		183.2	
10a	104.3		104.4		104.2		107.8		107.8	
11	204.8		204.0		206.4		203.9		204.0	
12	33.7	2.58	33.5	2.66	34.3	2.39	33.4	2.66	33.7	2.68
13	56.6	4.08	56.6	4.07	56.7	4.10	62.9	4.27	62.8	4.26
4-OH		12.53		12.49		12.61		12.37		12.36

* The Boltzmann weighted chemical shifts

** Boltzmann population

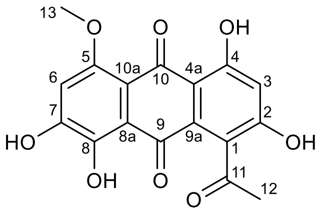

b

	**b**[*]		**b-conformer 1** (73%)[**]		**b-conformer 2** (25%)[**]		**b-conformer 3** (1.1%)[**]		**b-conformer 4** (0.5%)[**]	
position	$\delta_{C(calc)}$	$\delta_{H(calc)}$	$\delta_{C(calc)}$	$\delta_{H(calc)}$	$\delta_{C(calc)}$	$\delta_{H(calc)}$	$\delta_{C(calc)}$	$\delta_{H(calc)}$	$\delta_{C(calc)}$	$\delta_{H(calc)}$
1	121.7		123.6		116.0		123.5		116.0	
2	155.6		154.4		159.1		154.4		159.2	
3	108.1	6.90	107.8	6.86	109.0	7.01	107.5	6.85	108.7	6.99
4	162.4		162.0		163.5		161.9		163.5	
4a	110.9		110.5		112.0		110.5		111.9	
5	155.2		155.2		155.3		154.4		154.5	
6	104.1	7.27	104.0	7.27	104.4	7.28	104.5	7.00	104.6	7.00
7	151.3		151.2		151.7		151.0		151.5	
8	142.0		142.2		141.5		145.3		144.5	
8a	114.7		114.5		115.0		115.0		115.5	
9	185.7		185.3		186.9		185.1		186.7	
9a	132.1		130.9		135.8		131.3		136.3	
10	181.1		181.1		181.1		181.0		181.0	
10a	109.2		109.3		108.8		109.7		109.2	
11	204.7		204.2		205.9		204.2		205.9	
12	33.8	2.58	33.7	2.65	34.0	2.36	33.8	2.65	34.0	2.36
13	55.9	4.05	55.9	4.05	56.0	4.08	55.7	4.01	55.8	4.04
4-OH		13.51		13.49		13.57		13.49		13.56

[*] The Boltzmann weighted chemical shifts

[**] Boltzmann population
